# Three-Dimensional *In Vivo* Imaging of the Murine Liver: A Micro-Computed Tomography-Based Anatomical Study

**DOI:** 10.1371/journal.pone.0031179

**Published:** 2012-02-08

**Authors:** Teresa Fiebig, Hanne Boll, Giovanna Figueiredo, Hans Ulrich Kerl, Stefanie Nittka, Christoph Groden, Martin Kramer, Marc A. Brockmann

**Affiliations:** 1 Department of Neuroradiology, Medical Faculty Mannheim, University of Heidelberg, Mannheim, Germany; 2 Department of Clinical Chemistry, Medical Faculty Mannheim, University of Heidelberg, Mannheim, Germany; 3 Department of Veterinary Clinical Sciences, Small Animal Clinic, Justus-Liebig-University, Giessen, Germany; University of Birmingham, United Kingdom

## Abstract

Various murine models are currently used to study acute and chronic pathological processes of the liver, and the efficacy of novel therapeutic regimens. The increasing availability of high-resolution small animal imaging modalities presents researchers with the opportunity to precisely identify and describe pathological processes of the liver. To meet the demands, the objective of this study was to provide a three-dimensional illustration of the macroscopic anatomical location of the murine liver lobes and hepatic vessels using small animal imaging modalities. We analysed micro-CT images of the murine liver by integrating additional information from the published literature to develop comprehensive illustrations of the macroscopic anatomical features of the murine liver and hepatic vasculature. As a result, we provide updated three-dimensional illustrations of the macroscopic anatomy of the murine liver and hepatic vessels using micro-CT. The information presented here provides researchers working in the field of experimental liver disease with a comprehensive, easily accessable overview of the macroscopic anatomy of the murine liver.

## Introduction

Within the last twenty years the number of publications describing the use of mouse models has steadily increased. Animal models of human disease have become an integral part of virtually all areas of medical research. Consequently, various murine models of liver disease have been developed including models of inflammatory liver disease [1], [2], alcoholism [3], [4], portal hypertension [5], [6], parasitic infectious disease [7], [8], hepatectomy [9], as well as liver tumors and metastases [10], [11], [12], [13]. Most of these models are used for longitudinal monitoring of the disease process and to assess the effectiveness of novel therapeutic approaches. Methods that allow non-invasive longitudinal liver imaging include micro-computed tomography (micro-CT) [14], [15], magnetic resonance imaging (MRI) [16], ultrasound [17], as well as more sensitive techniques with lower resolutions, such as positron emission tomography (PET) [18], [19], [20] and fluorescence imaging [18]. The increased availability of high-resolution imaging modalities allow researchers the opportunity (and sometimes the need) to identify with precision the pathological processes at work, making knowledge of the macroscopic anatomical situation of the murine liver essential.

To gain a better understanding of the macroscopic anatomy of the murine liver and its associated microvasculature we compiled information from our own previous research in C57BL/6 mice using micro-CT and compared our observations to the literature.

## Materials and Methods

### Micro-CT

Micro-CT images were acquired as described recently [14], [21] using an industrial X-ray inspection system (Y.Fox; Yxlon International GmbH, Hannover, Germany) equipped with a transmission X-ray tube and a 12-bit direct digital flat bed detector (Varian PaxScan 2520; Varian, Palo Alto, CA, USA). The data used in our study was based upon previously performed liver imaging in ten male and female, 12–24 week old C57BL/6 mice with a body wheight between 30–35 g. After injection of a liver specific nanoparticular contrast agent (Viscover™ ExiTron™ nano; Miltenyi Biotec, Bergisch-Gladbach, Germany) or a blood pool contrast agent (Fenestra VC; Advanced Research Technologies Inc., Montréal, Canada), mice were anaesthetized, intubated and imaging of the vascular structures and of the liver was performed using the following scan parameters: tube current 80 kV; 75 µA; 190° rotation within 40 sec scan time and continuous image acquisition at 30 fps (frames per second). The resulting 1200 projections were reconstructed using the software provided by the manufacturer (Recon Studio; Yxlon International GmbH) using a filtered back projection algorithm with a matrix of 512×512×512. The voxel size ranged between 39×39×52 µm and 41×41×55 µm. Coronal, saggital, and axial reconstruction were prepared. All experiments were carried out after receiving the local ethics committee approval (Regierungspräsidium Karlsruhe; G-202/10). Institutional guidelines for animal welfare and experimental conduct were followed.

### Literature search analysis

Our literature search utilized, the search engines Medline, Google, and Vetseek [22], and also included veterinary anatomy books [23], [24], [25], [26], [27]. The search terms included: anatomy, mouse, mice, murine, liver, hepatic, hepatectomy, and imaging. To create comprehensive up-to-date illustrations of the murine liver, our review focused on gathering information that specifically detailed the shape, fixation and impressions of the liver, the location of the lobes, the adjacent perihepatic organs and the liver-related vessels.

## Results

### Literature search

Searching Medline we found four articles describing the macroscopic anatomy of the murine liver [9], [28], [29], [30]. In these four articles, three different terminologies for the anatomical description of the liver lobes were used and none of the descriptions conformed with *Nomina Anatomica Veterinaria* (NAV) which is the standard reference in veterinary science for anatomical terminology [31].

In addition to the journal articles available online, we identified seven books that included a description of the murine liver anatomy [23], [24], [25], [26], [27], [32]. Within the seven books, five different terminologies were used for the anatomical description of the liver. While four books did not refer to the NAV at all [23], [24], [25], [33], three books [26], [27], [32] used a simplified version of the of the NAV.

During our literature search we found no articles or books that comprehensively reviewed the murine liver anatomy with regard to the classical slice orientations used in small animal in vivo-imaging, nor did we find any articles that described the adjacent liver vessels in this context.

### Anatomy of the murine liver

#### Shape and impressions of the liver

The murine liver has a convex shaped cranial surface conforming to the vault of the diaphragm and a concave shaped caudal surface, which is adapted to the surface of the abdominal organs. While the gastric impression (*Impressio gastrica*) of the caudal surface of the left lateral liver lobe is caused by the stomach, the right kidney lies within the renal impression (*Impressio renalis*) located on the caudal surface of the caudate lobe. Other impressions of the liver are the duodenal (*Impressio duodenalis*), oesophageal (*Impressio esophagea*), and the jejunal impression (*Impressio jejunalis*), which are difficult to identify in small animal imaging.

#### Ligaments

**Figure 1:**
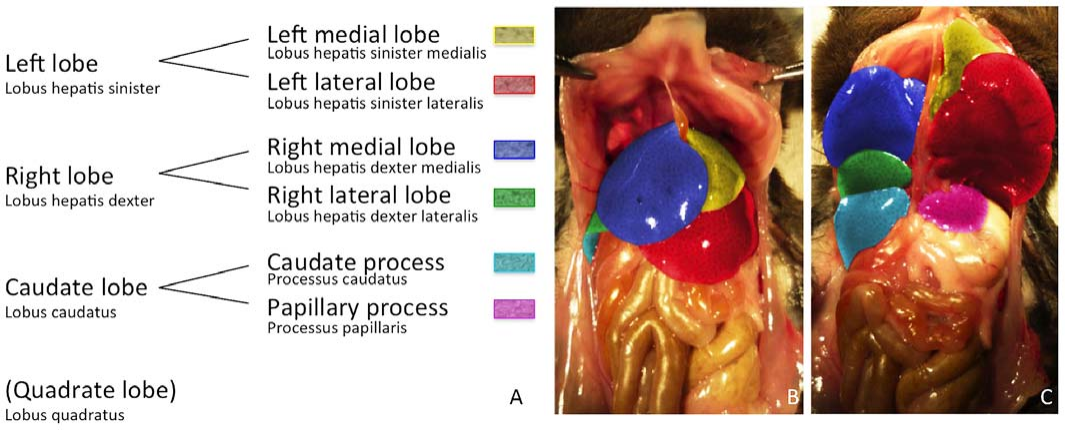
Illustrating the segmentation of the murine liver lobes according to the *Nomina Anatomica Veterinaria.* **A** Since in most animals we have not been able to identify the quadrate lobe in micro-CT imaging or in situ, the quadrate lobe was put in brackets and not color-coded. **B** and **C** are photographs of the liver of a C57BL/6J mouse (ventral view). In C the left and the right liver lobe have been folded cranially to reveal the liver lobes lying below. The single liver lobes have been consistently color-coded according to the schematic segmentation (**1A**) and to the related micro-CT figures 2, 3, 4 to simplify orientation.

The liver is fixed in place by several ligaments. On the dorsal surface the left medial liver lobe and the left lateral liver lobe are fixed to the diaphragm by the coronary ligament (*Ligamentum coronarium*) and the triangular ligament (*Ligamentum triangulare sinistrum*), respectively. According to the literature, mice have no ligaments stabilizing the right medial and right lateral liver lobe. On the right side, only the caudate process is fixed to the right kidney by the hepatorenal ligament (*Ligamentum hepatorenale*). Ventrally, the falciforme ligament (*Ligamentum falciforme*) containing the teres ligament (*Ligamentum teres hepatis*), is considered a relic of embryonic development rather than a ligament of fixation (Figure 1B and 1C).

#### Anatomy of the liver lobes

**Figure 2:**
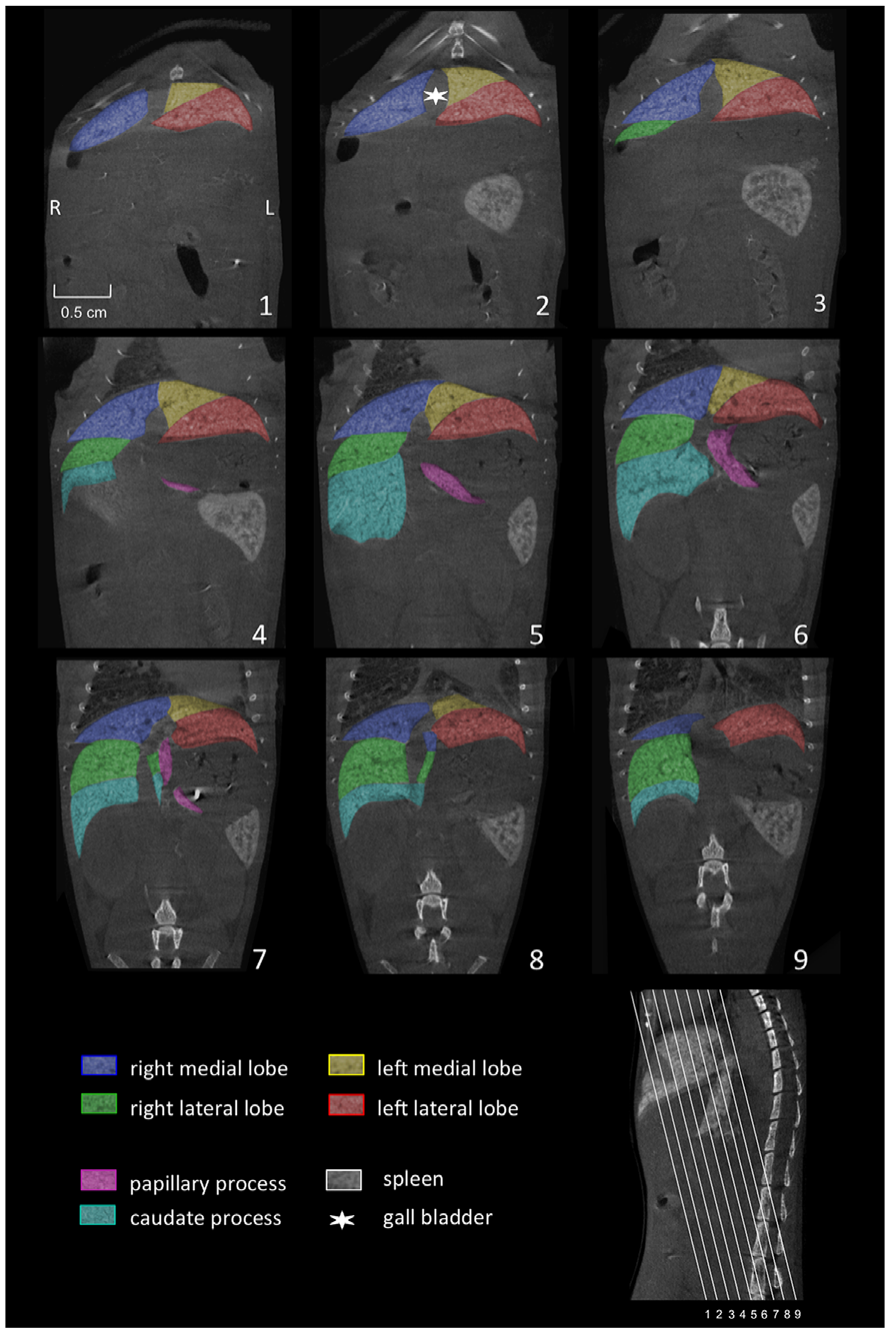
Coronally reconstructed micro-CT images. Imaging of the liver of a C57BL/6J mouse was performed 3 hours after i.v. injection of 100 µl of a nanoparticular contrast agent (ExiTron nano 12000). The color-coding of the single liver lobes corresponds to the color coding presented in Figure 1. The slice orientation in relation to the spine is shown in the lower right corner.

**Figure 3:**
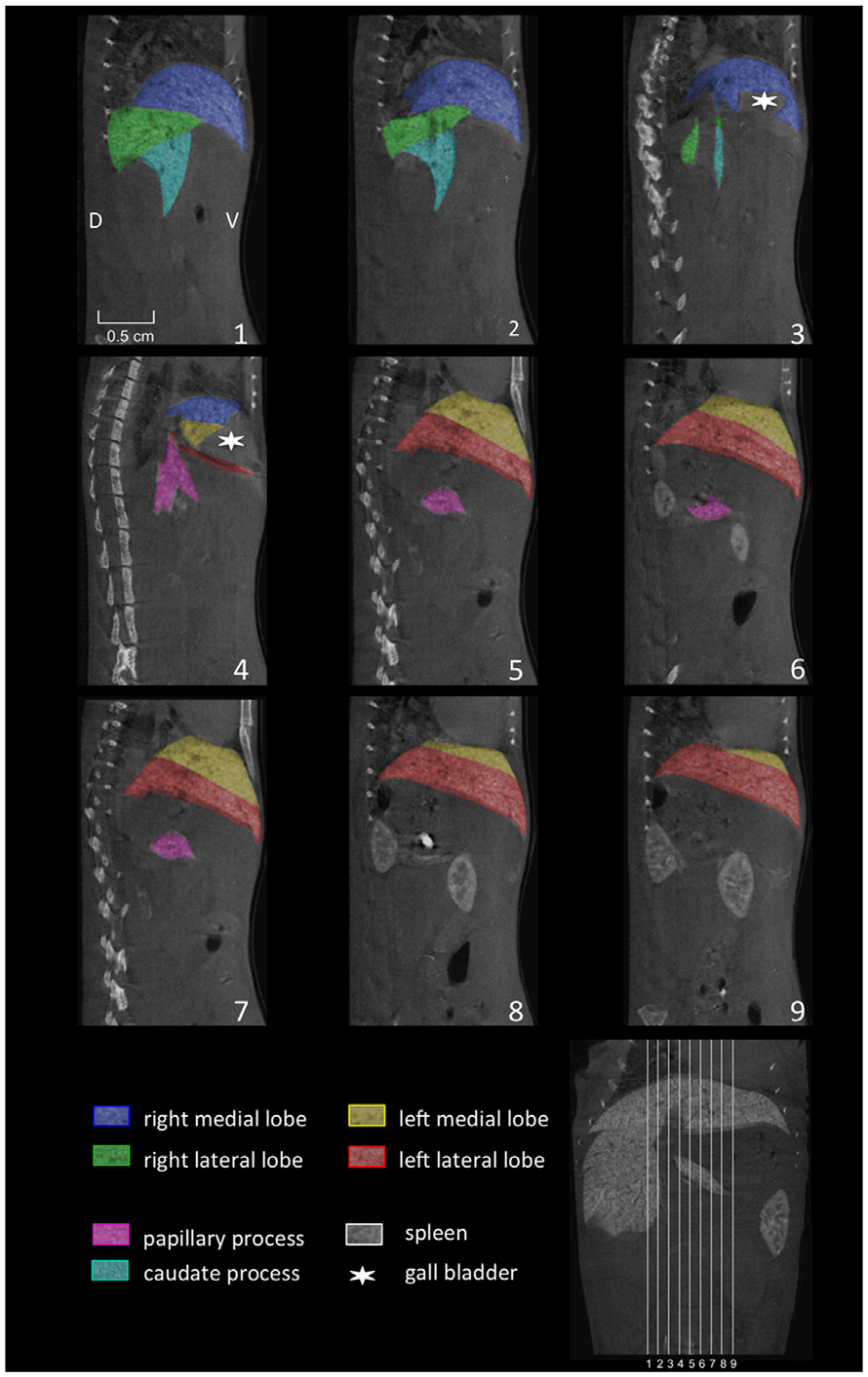
Saggital reconstructed micro-CT images. Micro-CT of the liver of a C57BL/6J mouse was performed 3 hours after injection of 100 µl of a nanoparticular contrast agent (ExiTron nano 12000). The color-coding of the single liver lobes corrsponds to the color coding presented in Figure 1. The slice orientation in relation to a coronal section is shown in the lower right corner.

**Figure 4:**
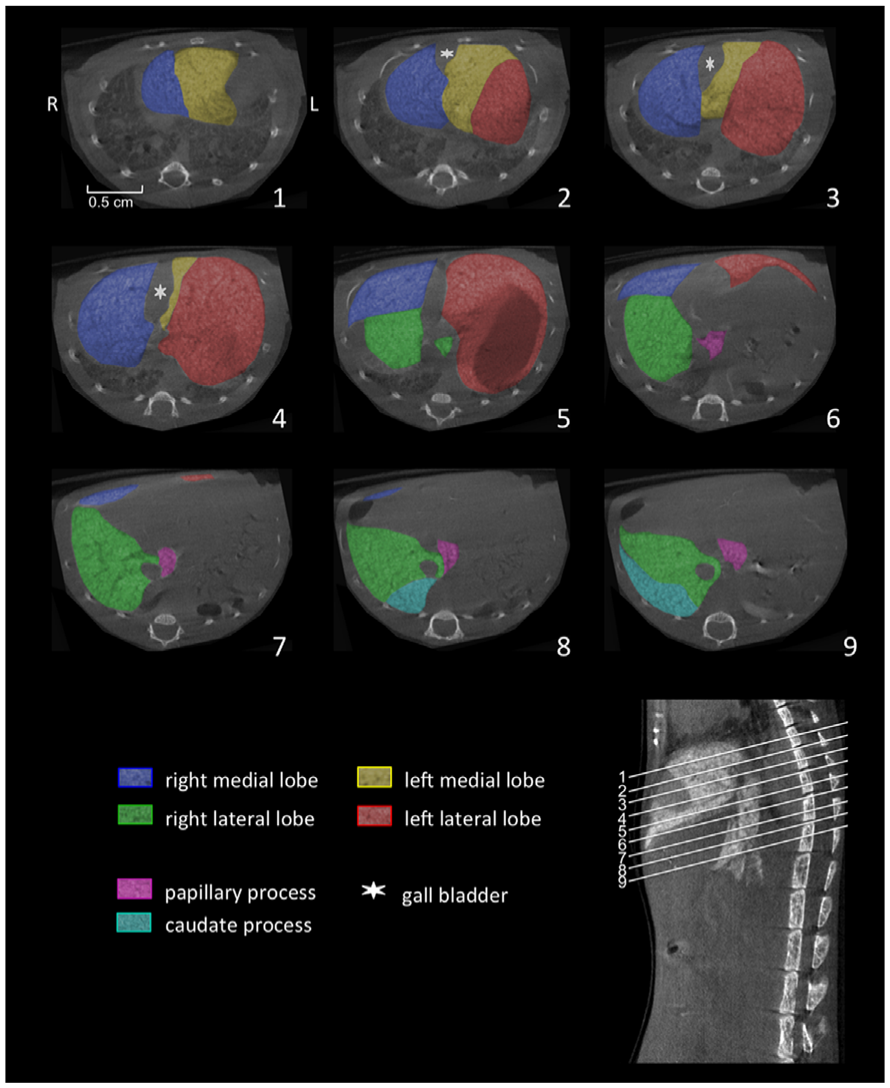
Axial reconstructed micro-CT images. Micro-CT of the liver of a C57BL/6J mouse was performed 3 hours after injection of a nanoparticular contrast agent (ExiTron nano). The color-coding of the single liver lobes corrsponds to the color coding presented in Figure 1. The orientation of the axial slices in relation to the spine is shown in the lower right corner.

According to the NAV, the liver is divided into four lobes that can be further subdivided, as schematically illustrated in Figure 1A, the following photographs (Figures 1B, 1C), and the reconstructions of micro-CT imaging in Figures 2, 3, 4.

The left liver lobe can be divided into the left medial lobe (yellow color-coding) and the left lateral lobe (red color-coding), with the smaller left medial lobe lying cranially and medially to the larger left lateral lobe (Figures 1, 2, 3, 4).

Similarly, the right liver lobe can be subdivided into the right medial lobe (blue color-coding), which is located directly below the diaphragm and lateral to the right side of the gall bladder, and the right lateral lobe (green color-coding), which is smaller and located more caudally than the right medial lobe.

The caudate lobe is subdivided into the larger caudate process (cyan color-coding) and the smaller papillary process (magenta color-coding). The larger caudate process lies directly caudal to the right lateral lobe and overlaps the right kidney ventrally and laterally. The papillary process in general is relatively small, and can be subdivided into two smaller parts (no specific nomenclature exists for these two subdivided parts) and is located between the stomach, the right lateral lobe, and the caudal caval vein.

Finally, the quadrate lobe is described in the NAV. This lobe is located at the medial edge of the left lateral lobe and is not further subdivided. While we have not been able to identify this small lobe of the liver macroscopically or with micro-CT images, other authors depicted the quadrate lobe in their schematic drawings without labeling it within the images [26], [32]. Only Popeskow et al. [27] correctly depicted and labeled the quadrate lobe in their schematic drawing. Although very small in mice, the quadrate lobe has been described as an independent lobe in the NAV, mainly because in many other animals the lobe is larger and therefore more easy to identify.

#### Anatomical variations

**Figure 5:**
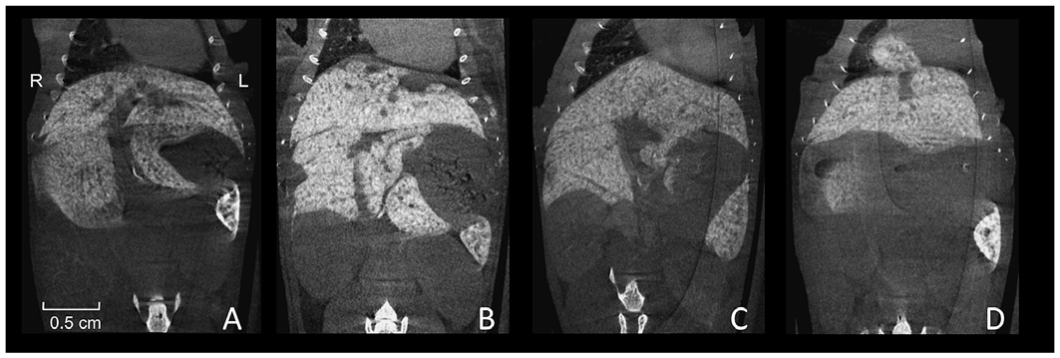
Liver variationes. **A–C** are comparable coronal sections of the murine liver illustrating the variability of the size of the papillary process of the caudate lobe. **D** shows transdiaphragmatic herniation of parts of the right medial liver lobe, which we incidentally discovered during our studies.

While anatomical variations of the liver do exist both within and between different mouse strains, to date, we did not evaluate these differences in-depth. However, we observed variations in our own measurements at the fusion of the two middle lobes. This variation has been described, by Rauch et al. [34] as fission rather than of fusion of the lobes especially among male animals. Other noted variations include the shape and size of the single liver lobes. Figure 5A–C illustrates the variability of the size of the papillar process.

### Perihepatic structures and hepatic vessels

To compliment the anatomical description of the liver we also gathered additional information on the anatomical relations of the adjacent perihepatic organs and liver-related vessels.

#### Organs adjacent to the liver

Only separated by the diaphragm, the heart and the lungs are cranial to the liver. While these organs are often easily distinguished from the liver, in one case we observed transdiaphragmatic herniation of the right medial liver lobe, which was clearly identifiable just after administration of a liver-specific contrast agent (Figure 5D).

**Figure 6:**
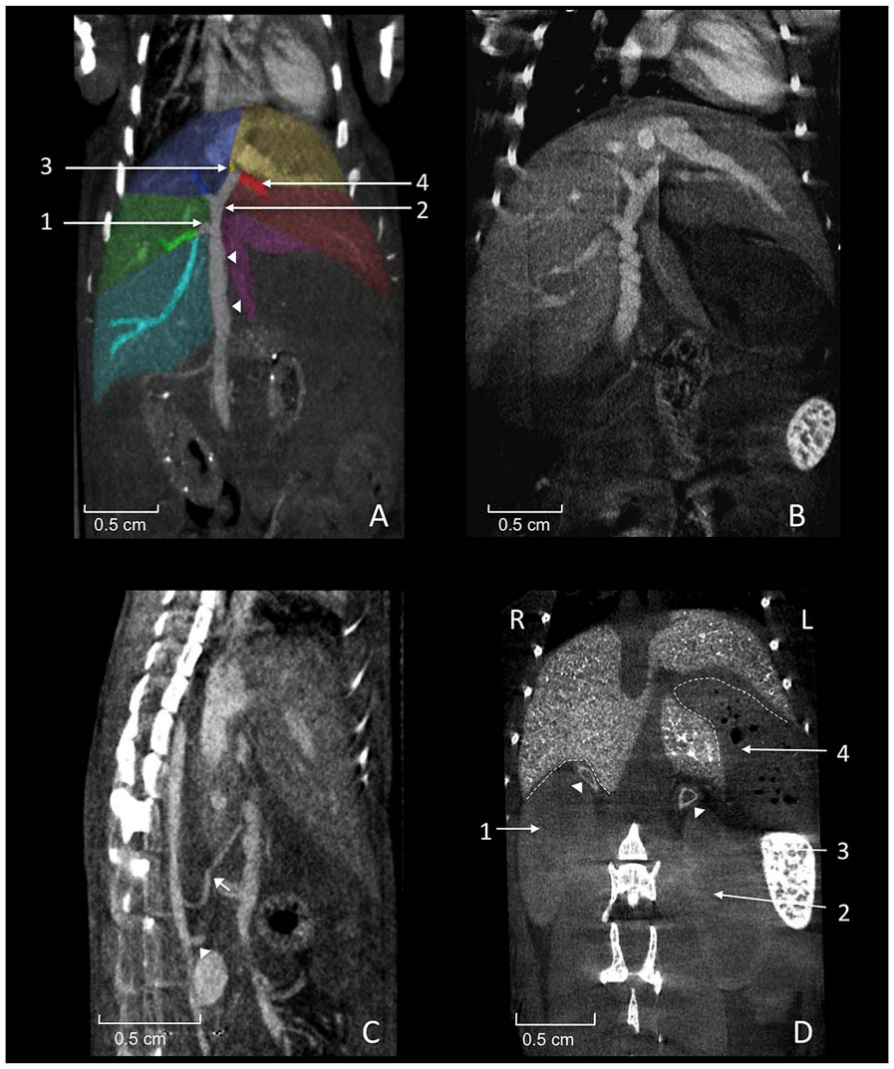
Perihepatic structures. **A** shows coronally reconstructed micro-CT images acquired 20 minutes after injection of 400 µl Fenestra VC. The portal vein (arrowheads) runs in a caudocranial direction and divides into single veins supplying the liver lobes with blood from the abdominal organs. The right branch (1; *Ramus dexter*) divides up into two non-specified branches supplying the right lateral lobe (colored green) and the right caudate process (colored cyan). The left branch (2; *Ramus sinister*) divides into the umbillical part (3; *Pars umbillicalis*) and the transversal part (4; *Pars transversalis*) draining the left medial lobe and the left lateral lobe, respectively. The vein supplying the right medial liver lobe (colored blue) is not classified in the NAV. Spontaneous peristaltic movement of the portal vein is known to result in a corkscrew-like appearance of the vessel as shown in **B**. The liver is supplied with arterial blood from the coeliac artery (arrow) arising from the aorta cranial of the cranial mesenteric artery (arrowhead), as shown in **C**. The portal vein collecting blood from smaller abdominal vessels can be spotted directly ventral to the hepatic artery. **D** shows a coronally reconstructed micro-CT, 21 days after injection of 100 µl Exitron nano 12000 showing relevant organs adjacent to the liver. The right kidney (1), the left kidney (2), the spleen (3), the stomach (4; containing air bubbles), and the adrenal glands (arrow heads). The dotted lines indicate the renal impression of the liver on the right side and the gastric impression on the left side.

The half-moon shaped spleen can be found caudal to the liver, kidneys, adrenal glands, and stomach (Figure 6D). As mentioned earlier in the manuscript, the right kidney lies a little more cranially towards the left kidney, reaches the liver surface and lies within the renal impression (*Impressio renalis)* of the liver. Between the medial cranial pole of the kidneys and the liver the triangularly shaped adrenal glands are located on both sides (Figure 6D). On the left side of the abdomen the stomach lies within the gastric impression (*Imressio gastrica*) adjacent to the surface of the left lateral lobe, while the spleen is located between the left kidney, the stomach, and left abdominal wall.

#### Hepatic artery

The coeliac artery (*Arteria coeliaca*; Figure 6C) arises from the abdominal aorta before the cranial mesenteric artery (*A. mesenterica cranialis*, in humans the superior mesenteric artery) and among others branches into the hepatic artery which runs cranially. Due to the limitations of in vivo micro-CT of the hepatic artery we have been able to identify the proximal hepatic artery, but cannot trace this vessel into the liver. However, the branching pattern of the hepatic artery has been reported to correspond with the branching of the portal vein and the billiary ducts [35], [36].

#### Portal vein

The portal vein drains blood from the gastrointestinal tract and spleen to the liver. The portal vein divides into the right branch (*Ramus dexter*) draining into the caudate process and the right lateral lobe (Figure 6A), and the left branch (*Ramus sinister*). The left branch provides blood-flow into the right medial lobe and divides into the umbilical part (*Pars umbillicalis*) for the left medial lobe and the transversal part (*Pars transversalis*) for the left lateral lobe. The NAV provides no specific nomenclature for the small branch providing blood supply to the papillary process.

#### Hepatic veins

**Figure 7:**
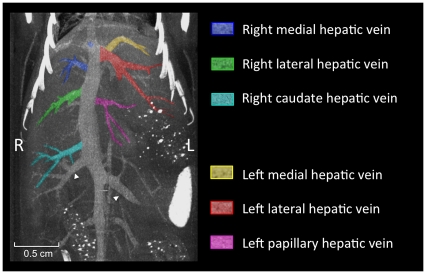
Hepatic veins. Maximum intensity projection (MIP) of micro-CT images of the murine hepatic veins 20 minutes after injection of 400 µl Fenestra VC. The color coding of the draining veins corresponds to the color coding used for the liver lobes before. The renal veins are indicated by arrowheads.

The hepatic veins are not well described in the NAV. Only a left, a right, and a medial hepatic vein are described. However, using in vivo micro-CT angiography, more veins draining into the inferior vena cava can be identified (Figure 7). To address this problem we referred to the NAV to describe the side of the vein, and added information on the draining territory (i.e. lateral or medial), which matches the nomenclature of the right and the left liver lobe. For the processes of the caudate lobe we extended the name of the vein by adding the name of the drained lobe (i.e. the left papillary hepatic vein).

The most caudally located vein draining the caudate process is the right caudate hepatic vein. The next most cranially located branch on the right side draining the right medial lobe is the right medial hepatic vein, while the right lateral lobe is drained by the right lateral hepatic vein.

The left lateral liver lobe is drained by the left lateral hepatic vein. The left medial lobe is drained by the left medial hepatic vein, that drains into the left lateral hepatic vein. The papillary process of the caudate lobe is drained by a small, separate branch which would be termed the left papillary hepatic vein.

### Billiary tract

Using contrast agents that are not being excreted via the biliary tract, the only clearly identifiable part of the billiary system in micro-CT images is the gall bladder, which is depicted in Figures 2, 3, 4.

## Discussion

Searching the literature we found a limited number of publications sparsely describing the macroscopic anatomy of the murine liver [23], [24], [25], [26], [27], [30]. In addition, we found no existing publications referring to the increasingly available imaging modalities. Within the publications that described the macroscopic anatomy of the murine liver we noted a lack of consistency in nomenclature [30], [35]. Reasons for the inconsistency in terminology includes the alternating use of Latin and English terminology and, more problematic, inconsistency in nomenclature of the structures between different authors [9], [24], [26], [27], [37]. For example, while the left liver lobe (according to the NAV) was correctly divided into the left lateral and left medial liver lobe by Popesko et al. [27], other authors divided the left liver lobe into the left lateral lobe and the left part of the medial lobe [24], [25], [28], [30]. Likewise, the lobes of the right side of the murine liver have been found to be accompanied by different adjectives including “posterior”, “anterior“, “inferior“, “superior”, “upper” and “lower” by different authors [9], [24], [28], [30], [35], [37], [38], again creating confusion.

The most serious differences exist in the terminology of the quadrate lobe and the caudate lobe (with the latter one according to the NAV being subdivided into the papillary process and the caudate process). For example, some authors described the caudate process (of the caudate lobe) as the caudate lobe [26], [27] or the right lower lobe [9], while other authors named the papillary process as the caudate [23], [24], [25], [28], [30] or the omental lobe [9], due to its anatomical surroundings.

To overcome the problems that may arise from the inconsistent designation of the liver lobes we recommend using the nomenclature in accordance with the *Nomina Anatomica Veterinaria*, which was introduced in 1955 and since then has continuously been updated by an international group of veterinarians. Regarding the murine liver lobes we found the NAV to serve almost perfectly (with the only exception that we found it difficult to identify the quadrate lobe). However, there were some problems in the most recent edition of the NAV from 2005 [31]. For example, as noted in the results section, the nomenclature of the draining veins of the liver, as described in the NAV, are not easily transferrable to mice.

**Figure 8:**
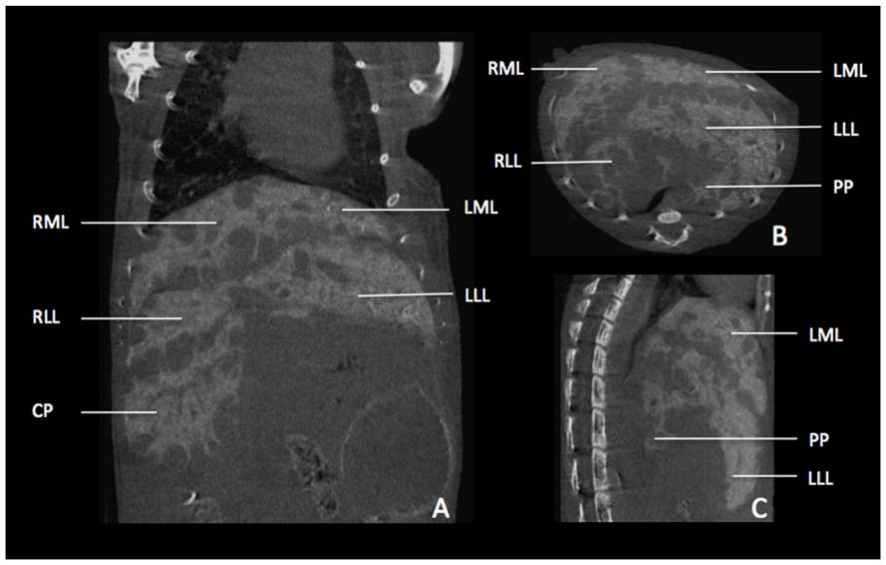
Illustrative micro-CT images of liver metatstases in a mouse. **A–C** Images of liver metastases were acquired 2 hours after i.v. injection of 100 µl of a contrast agent (Viscover Exitron nano 6000; Miltenyi Biotec, Bergisch-Gladbach, Germany) in a coronal (A), transversal (B), and saggital (C) slice orientation. With regard to liver anatomy, we found metastases to develop predominantly under the liver capsule adjacent to the fissures between the liver lobes. While the left lateral lobe (LLL) shows no metastases on its caudal edge in this slice, there are plenty on its cranial edge, next to the left medial lobe (LML). The lobes on the right side (right medial lobe (RML), right lateral lobe (RLL) and the caudate process (CP)) show (besides the subcapsular growth pattern) metastases within the liver parenchyma. Due to tumor growth the papillary process depicted in B and C lost its typical shape (compare with figs. 2, 3, 4).

Photographs and schematic drawings are primarily used in virtually all other publications dealing with the anatomy of the murine liver. Although different three-dimensional mouse atlases are available in book form [23], [27], [33] and online [39], [40], the precision of the anatomical description of the liver isn't as comprehensive as presented here. To better represent the increasing use of small animal imaging modalities in liver research, we present the anatomy of the murine liver using the three typical radiological cut planes implemented in tomographic imaging which allows for a faster and better orientation of the liver relative to other adjacent anatomical structures. This might be helpful for anatomical studies or to reveal strain differences, but also for animal models of human disease as described in the introductory section and as exemplarily shown in figure 8.

While our study aimed at providing a general overview of the macroscopic anatomy of the murine liver, we did not investigate differences related to mouse strain, gender, weight, or age. These differences, however, have to be considered when trying to refer to the anatomical descriptions presented in our manuscript.

### Conclusion

The provided three-dimensional illustrations of the macroscopic anatomy of the murine liver using micro-CT may promote clarity and precision among scientists and veterinarians working in the field of liver research and may be helpful as a reference for future experimental research in this field.
